# Improving Tannery Wastewater Treatments Using an Additional Microbial Treatment with a Bacterial–Fungal Consortium

**DOI:** 10.3390/biology12121507

**Published:** 2023-12-08

**Authors:** Fuad Ameen

**Affiliations:** Department of Botany & Microbiology, College of Science, King Saud University, Riyadh 11451, Saudi Arabia; fuadameen@ksu.edu.sa

**Keywords:** tannery effluent, collagen, hydroxyproline assay, enzymes, microbial consortium

## Abstract

**Simple Summary:**

Several large tanneries operate in Saudi Arabia. The tanneries produce more than 15,000 pieces of leather per day and 200 metric tons of wastewater per day. Leather manufacturing is a highly polluting activity throughout the world, and in many places, it is not known how efficient the wastewater treatments in the factories are. This was found to be the case in Saudi Arabia, where we revealed insufficient treatments in all four of the factories that we studied. We propose here an additional biological treatment using a bacterial–fungal consortium. Bacteria and fungi were isolated from tannery wastewaters, and their collagenase and gelatinase activities were measured to discover their potential to clean tannery wastewater. Different single bacteria and fungi and their consortia were used in the experiment to find the most suitable species for the treatment. The treatment appeared to be efficient, reducing almost all parameters to below the environmental regulation limit for wastewater discharge to the environment in Saudi Arabia.

**Abstract:**

Environmental pollutants such as toxic heavy metals and oxygen-demanding solids are generated by leather manufacturing. In most tanneries, wastewaters are treated with physico-chemical methods but overly high levels of pollutants remain in surface waters. The efficiency of tanning wastewater treatment with conventional techniques was evaluated in four tanneries in Saudi Arabia. It was observed that the wastewaters contained high amounts of pollutants, needing further treatment. We isolated microorganisms from the wastewaters and carried out experiments to treat the effluents with different bacteria, fungi, and their consortia. We hypothesized that a consortium of microorganisms is more efficient than the single microorganisms in the consortium. The efficiency of five single bacterial and five fungal species from different genera was tested. In a consortium experiment, the efficiency of nine bacterial–fungal consortia was studied. The bacterium *Corynebacterium glutamicum* and the fungus *Acremonium* sp. were the most efficient in the single-microbe treatment. In the consortium treatment, the consortium of these two was the most efficient at treating the effluent. The factory wastewater treatment reduced total dissolved solids (TDS) from 1885 mg/L to 880 mg/L. *C. glutamicum* treatment reduced TDS to 150 mg/L and *Acremonium* sp. to 140 mg/L. The consortium of these two reduced TDS further to 80 mg/L. Moreover, the factory treatment reduced BOD from 943 mg/L to 440 mg/L, *C. glutamicum* to 75 mg/L, and *Acremonium* sp. 70 mg/L. The consortium reduced BOD further to 20 mg/L. The total heavy-metal concentration (Cd, Cr, Cu, Mn, and Pb) was reduced by the factory treatment from 43 μg/L to 26 μg/L and by the consortium to 0.2 μg/L. The collagen concentration that was studied using hydroxyproline assay decreased from 120 mg/L to 39 mg/L. It was shown that the consortium of the bacterium *C. glutamicum* and the fungus *Acremonium* sp. was more efficient in reducing the pollutants than the single species. The consortium reduced almost all parameters to below the environmental regulation limit for wastewater discharge to the environment in Saudi Arabia. The consortium should be studied further as an additional treatment to the existing conventional tannery wastewater treatments.

## 1. Introduction

Leather processing in tanneries generate wastewaters that contain polluting organic and inorganic compounds. The pollutants originate from both animal skin and chemicals used in the tanning process. Animal skin particles consume oxygen and release nutrients when released into surface waters [1]. The chemicals include heavy metals, particularly chromium (Cr) [2,3]. In addition, pesticides, salts, and acids are found in high concentrations [4,5]. The phyto- and genotoxicity of these chemicals has been shown in numerous studies [1,6,7,8,9]. As much as 40% of the environmental heavy metal load has been found to originate from tanning [10]. Thus, the wastewaters of the tanneries need to be treated before being released into the environment [11].

In tanneries, the wastewaters are usually treated with different systems. Conventional treatment systems include coagulation, flocculation, sedimentation, filtration, and biological treatments by activated sludge and its variants [12]. However, these techniques are often not efficient in removing pollutants. In particular, the high salinity and turbidity of the wastewater make the treatments challenging [13,14]. To improve the efficiency, advanced treatments such as electro-chemical methods, advanced oxidation processes, microfiltration, and reverse osmosis are increasingly used [15].

Heavy metals, particularly Cr, in tannery wastewaters are challenging pollutants due to their high toxicity and elementary nature. Different combinations of techniques such as membrane bioreactor–photoelectron oxidation have shown their efficiency and are increasingly being studied. Several other combinations of techniques have been suggested in a recent review by [12]. These advanced techniques are efficient in removing Cr, while adsorption techniques have also shown their efficiency. Inorganic adsorbents in combination with local plant species in constructed wetland have been found to remove tannery pollutants efficiently [16]. Organic adsorbents such as waste tea leaves have also been presented as an efficient, economic, and environmentally sustainable adsorbent of Cr and other heavy metals in tannery wastewaters [17]. Certain microorganisms have the capacity to accumulate heavy metals within their cells (bioaccumulation) or on their surfaces (biosorption). This process can be utilized in reducing the concentration of heavy metals in the wastewater. Microbial adsorption of heavy metals has been extensively used, for instance, in different remediation practices [18,19]. The efficiency of microbes has been shown in many different applications for decades [20]. Biological treatments are often low-cost and sustainable, operating at relatively low energy consumption levels.

Biological treatment systems feed on organic pollutants, breaking them down into simpler, less harmful substances such as carbon dioxide, water, and biomass. In tannery wastewaters, animal skin residues are major pollutants containing fats, gelatins, and proteins [21]. While the proteins in tannery wastewaters, namely collagen and its degraded form gelatin, are valuable in many applications, they are harmful in wastewaters because they consume oxygen and release nutrients into surface waters [22]. The protein collagen is the major skin component [21]. Thus, the bacteria and fungi in cleaning tannery wastewaters must be able to degrade collagen and gelatin. Thus, the most suitable microorganisms can be found by measuring their enzyme activities, namely collagenase and gelatinase activities. For instance, the bacterial genera *Bacillus*, *Streptomyces*, and *Pseudomonas* and the fungal genera *Aspergillus*, *Fusarium*, and *Cladosporium* have been reported to remove organic pollutants in tannery wastewater [23]. Because the collagen should be degraded in the treatment, its concentration should be measured after the treatment to assess its efficiency.

Different consortia of microbes, often *Bacillus* sp. and *Pseudomonas* sp., have been shown to be more efficient in removing many kinds of pollutants when compared to single microbes [24]. The consortium of *B. subtilis* and *Acremonium* sp. has been shown to be more tolerant of heavy metals than the single species in the consortium [25]. Tannery wastewaters have also been treated efficiently with different consortia [26,27,28]. However, in the case of tannery wastewaters, comparative studies between the consortia and the single species are lacking. Thus, more information is needed on whether the wastewaters already treated in a tannery can be cleaned further with certain single microbes or their consortia. The hypothesis is that a consortium of certain microorganisms is more efficient than the single bacteria or fungi in the consortium. The novel approach of this study is to search a selection of microorganisms with a high capacity to degrade the specific organic contaminants in tannery wastewater. It is also important to use local, native microorganisms adapted to the existing conditions with no need to introduce possible harmful non-native species.

Several large tanneries operate in Saudi Arabia. The tanneries produce more than 15,000 pieces of leather per day and produce 200 metric tons of wastewater per day [7,29]. Wastewater treatment systems are in operation in these tanneries, but it is not known how efficient they are. The aims of this study were, firstly, to study the efficiency of the current wastewater treatments in Saudi Arabia and, secondly, to find bacterial and fungal species or their consortia that can be used to remove pollutants from tannery effluents. To find the most suitable microorganisms, bacteria and fungi were isolated from tannery wastewaters, and their collagenase and gelatinase activities were measured to discover their potential to clean tannery wastewater. Two experiments to treat the factory-treated tannery wastewater were carried out. In the first experiment, different single bacteria and fungi were tested, and in the second experiment, their consortia were used to test the hypothesis and to find the most suitable species for the tannery wastewater treatment.

## 2. Materials and Methods

### 2.1. Sample Collection

Tannery effluents were collected from four locations in Riyadh, Saudi Arabia: Modern Leather Factory, Al Jabreen Leather Factory, Al-Ahli Leather Factory, and Safi Universal for Leather Tanning. Each factory operates with the same sewage treatment processes that start with dissolved air flotation (DAF) and continue with biological treatment-activated sludge followed by pressure filtration and final carbon filtration. The samples were collected before (original wastewater) and after the sewage treatment of the factory (factory-treated wastewater) during the summer season of 2022. The collected samples were brought to the laboratory and stored at 4 °C.

### 2.2. Physical and Chemical Properties

The pollution in the tannery wastewaters (original and factory-treated) was evaluated with different pollution parameters: seven physico-chemical properties and five heavy-metal concentrations. Total dissolved solids (TDS) were measured using a TDS tester (AquaDart Digital, West Yorkshire, UK), electric conductivity using an electric conductivity meter (U-Tech, Kingston, Jamaica), and turbidity using an electronic turbidity meter (Hach turbidimeter, Loveland, CO, USA). Biological and chemical oxygen demand were analyzed using a BOD meter (BD600) and COD meter (Lovibond^®^, Wiltshire, UK), respectively. Dissolved CO_2_ concentration was measured using a dissolved CO_2_ sensor (MG811), and pH using a digital pH meter. Manganese (Mn), chromium (Cr), cadmium (Cd), copper (Cu), and lead (Pb) concentrations were measured using an atomic absorption spectrometer (Analyst 800, Perkin-Elmer, Shelton, CT, USA) [30].

### 2.3. Isolation and Identification of Microorganisms

The original tannery wastewater before the factory sewage treatment was serially diluted, and 1 mL was spread onto potato dextrose agar (PDA) plates and nutrient agar plates (NA) (HiMedia, Thane, India). The plates were incubated at 28 °C for 7 days and 2 days, respectively. Different bacteria and fungi were isolated based on morphological characteristics, making five bacterial and five fungal isolates in total. The isolates were subcultured on PDA and NA plates and used for further studies. The isolates were transferred to sterile Eppendorf tubes containing 1 mL of 30% (*v*/*v*) sterile glycerol, incubated at 28 °C for 5 days, and then maintained at −20 °C.

The bacterial isolates were incubated in nutrient agar broth (HiMedia, Thane, India) at room temperature in an orbital shaker. The bacterial DNA was extracted using a HiPer extraction kit following the manufacturer’s instructions. Bacterial 16S rRNA was amplified using the primers 27F and 1492R [31]. The PCR reaction mixture (50 µL) contained 2 µL (50–100 ng) of DNA, 1x reaction buffer (TrisKCl-MgCl2), 2 mM MgCl_2_, 0.2 mM dNTP, 1 µM of each primer, and Taq polymerase (5 U/µL, Fermentas, Waltham, MA, USA). The PCR temperature cycling conditions were as follows: initial denaturation at 94 °C for 2 min; 30 cycles of denaturation at 94 °C for 1 min, annealing at 55 °C for 2 min, and elongation at 72 °C for 2 min followed by extension at 72 °C for 5 min.

The fungal isolates were inoculated into PDB (potato dextrose broth) and incubated in an orbital shaker at room temperature. The DNA was extracted using a HiPurA fungal DNA isolation kit following the manufacturer’s instructions. PCR amplification was performed as described by [32]. ITS1 (5′-TCCGTAGGTGAACCTGCGG-3′) and ITS 4 (5′-TCCTCCGCTTATTGATATGC-3′) primers were used. The PCR conditions were as follows: initial denaturation at 95 °C for 5 min, 35 cycles of denaturation at 94 °C for 1 min, annealing at 55 °C for 3 s, extension at 72 °C for 1 min, followed by a 10 min final extension step at 72 °C.

A BigDye terminator sequence kit (Applied Biosystems, Waltham, MA, USA) was used for the sequencing. The sequences were identified with NCBI BLAST + 2.8.1 software, with additional alignments using Ugene and the T-Coffee algorithm (https://www.ebi.ac.uk/, EMBL-EBI, Cambridgeshire, UK). The phylogenetic tree was generated using the interactive tree tool iTOL (http://itol.embl.de/index.shtml).

### 2.4. Collagenase and Gelatinase Activities

The bacterial and fungal isolates were analyzed for their collagenase and gelatinase activities [33]. Gelatinase activity was measured on gelatin DEV agar plates (HiMedia, Thane, India) after inoculation and incubation at 28 °C (24–48 h for bacteria and 5–7 days for fungi). The plate was flooded with saturated ammonium sulphate solution, and the clearance zone was measured. For collagenase activity [34], 1% collagenase (HiMedia, Thane, India) and 2% agar plates were prepared. After the incubation described above, the plate was flooded with mercuric chloride precipitation reagent (15 mg/L HgCl_2_ + 20 mL HCl in 00 mL distilled water), and the clearance zone was measured.

### 2.5. Tannery Effluent Treatments

The factory-treated wastewater from Al-Ahli Leather Factory was used in two different treatments as three replicates. In the first treatment, the microbial species were used separately as three replicates. This single treatment consisted of five bacterial and five fungal isolates. The NB and PDB media (100 mL) were inoculated with bacteria and fungi, respectively, and incubated at 37 °C (1 day for bacteria, 4 days for fungi). After the incubation, the single treatment was carried out by adding the organism medium (100 mL) to 5 L of tannery wastewater in a 20 L glass tank with sterilized wheat straw pieces (5 kg) and incubated for 15 days at 37 °C with agitation. After the treatment, the pollution parameters were measured as described above.

The second treatment was the consortium treatment consisting of nine different microbial consortia. First, the nine microbial consortia consisting of one bacterial (NA-50 mL) and one fungal (PDB-50 mL) species were incubated at 37 °C for 2 days. After the preliminary incubation, the consortium treatment was carried out by incubating the organisms (50 mL bacteria + 50 mL fungi) in tanks as described above.

#### 2.5.1. Collagen

The collagen concentration was analyzed from the factory-treated wastewater of Al-Ahli and the consortia-treated tannery effluents using a hydroxyproline assay kit (Chondrex, Inc., Woodinville, WA, USA) according to the manufacturer’s instructions. The wastewater (100 µL) was added to 100 µL of concentrated HCl solution and incubated at 120 °C for 24 h. An aliquot of 10 µL of each sample was added to separate wells, then 100 µL of the 1X chloramine T solution was added and incubated at room temperature for 20 min. Then, 100 µL of 1X DMAB (p-dimethylaminobenzaldehyde) solution was added to each well and incubated at 60 °C for 30 min. The optical density (OD) was measured with a spectrophotometer at 530 nm. Hydroxyproline concentration was calculated with the following equation:

Hydroxyproline (µg/mL) = Hydroxyproline (µg/mL) × (Sample Volume (mL) + HCl Volume (mL))

#### 2.5.2. Sample Volume (mL)

Hydroxyproline concentration was converted to collagen concentration with the following equation:

$$\frac{\mathrm{Collagen}(µg/\mathrm{mL})=\mathrm{Hydroxyproline}(µg/\mathrm{mL})\times 100}{13.5}$$

## 3. Results

### 3.1. Bacterial and Fungal Species with Collagenase and Gelatinase Activities

The bacterial isolates were identified as Pseudomonas fluorescens, Bacillus subtilis, Acinetobacter baumannii, Enterobacter cloacae, and Corynebacterium glutamicum (Table 1).

**Figure 1:**
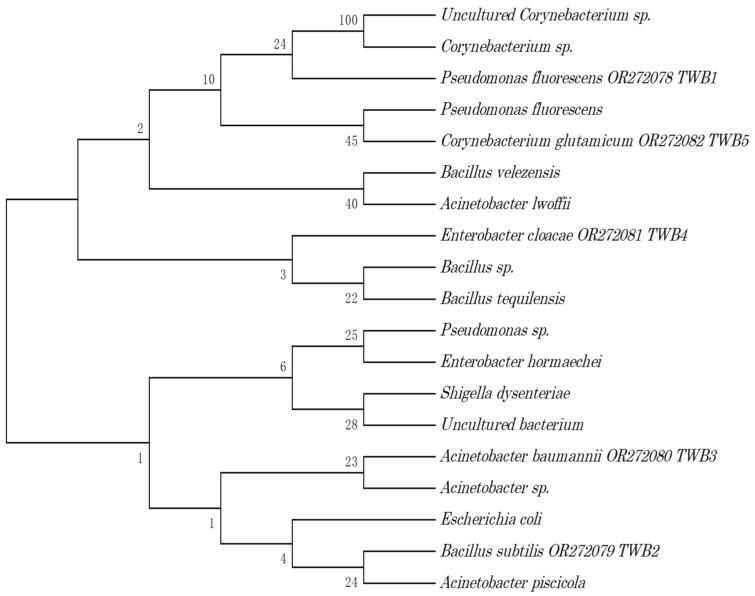
Phylogenetic tree of the bacteria TWB1–TWB5 isolated from tannery effluents.

**Figure 2:**
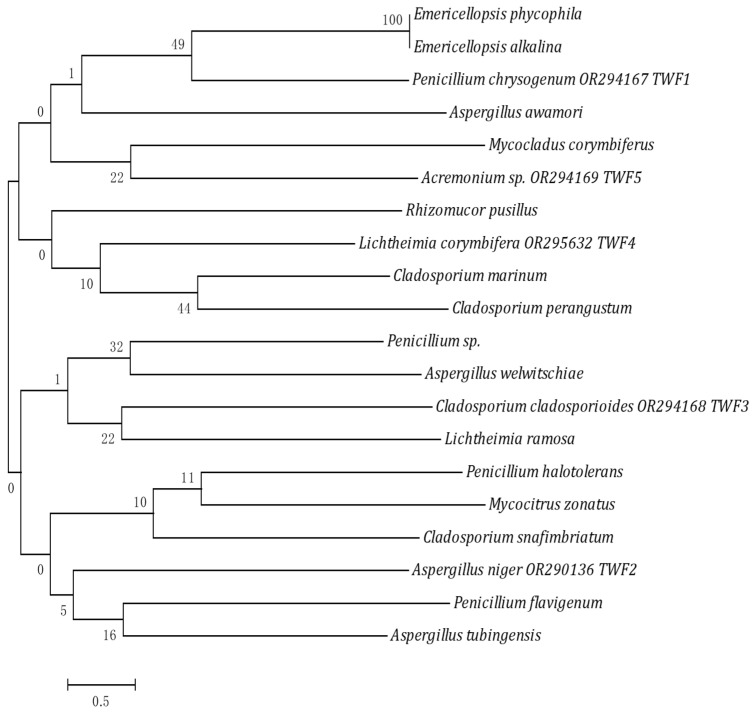
Phylogenetic tree of the fungi TWF1–TWF5 isolated from tannery effluents.

The identified fungal isolates were *Penicillium chrysogenum*, *Aspergillus niger*, *Cladosporium cladosporioides*, *Lichtheimia corymbifera*, and *Acremonium* sp. The phylogenetic trees are presented in Figure 1 and Figure 2.

**Figure 3:**
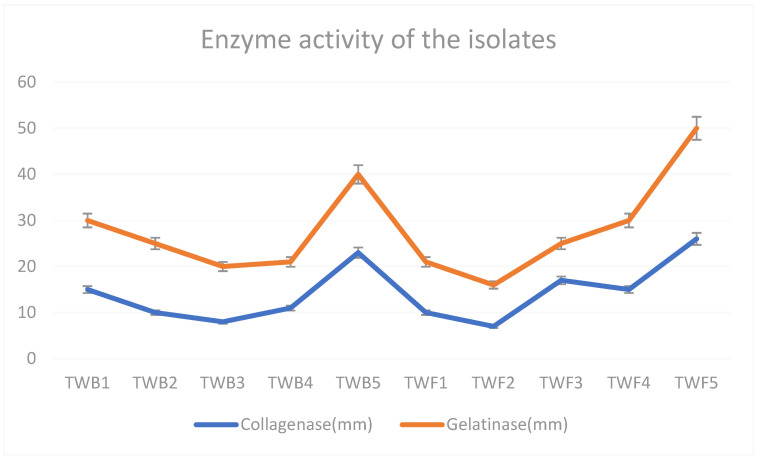
Collagenase and gelatinase activities measured as the zone of clearance (mm) (error bars for SD, *n* = 3) of the bacteria (TWB1–TWB5) and fungi (TWF1–TWF5) isolated from five different tannery effluents. See species names in Table 1.

Collagenase and gelatinase activities were the highest for *C. glutamicum* (respective zones of clearance: 40 mm and 23 mm) and *Acremonium* sp. (50 mm and 26 mm) (Figure 3).

### 3.2. Tannery Effluents

**Figure 4:**
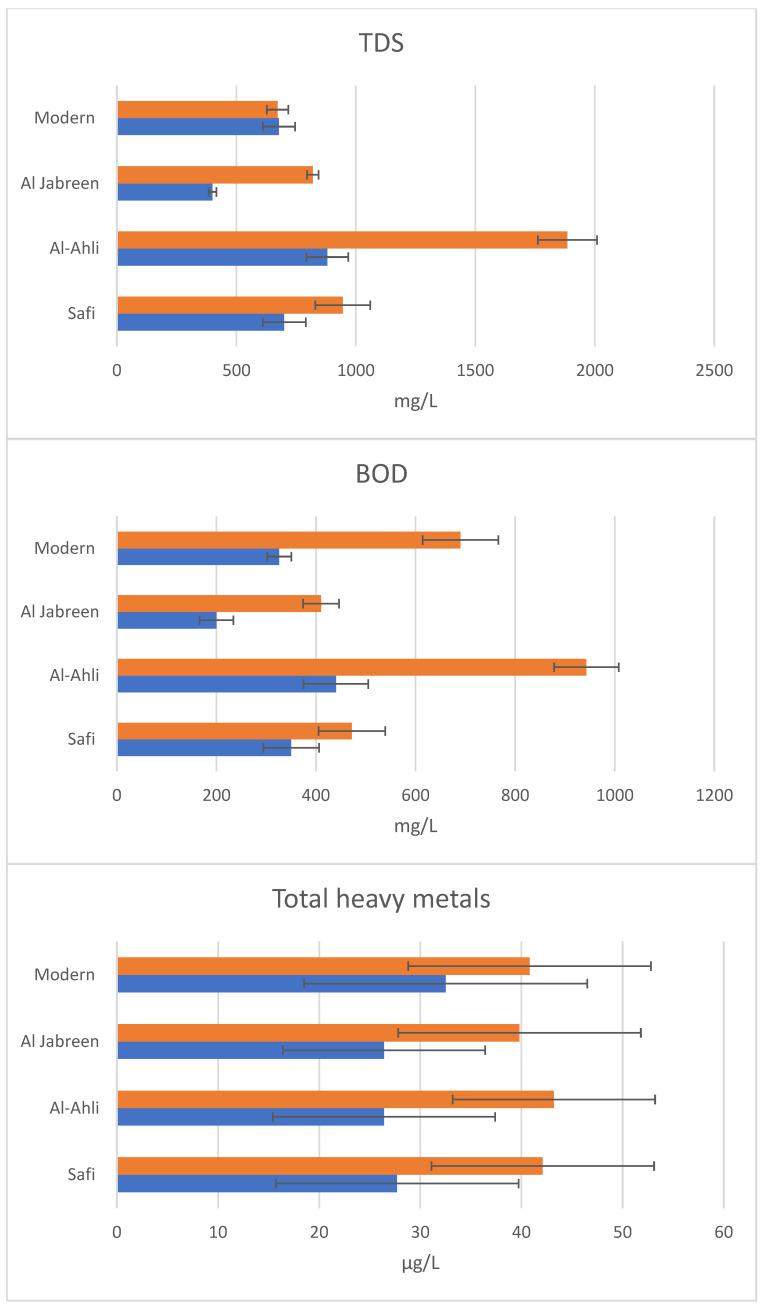
Pollution parameters (concentrations of TDS, BOD, total heavy metals; error bars for SD, *n* = 3) of the original wastewaters (orange) and factory-treated wastewaters (blue) from four different tanneries (y-axis).

Each leather factory’s original wastewater had relatively high values of the pollution parameters. Their individual factory sewage treatment efficiencies varied. The highest original wastewater pollution was observed at Al-Ahli (Figure 4).

The factory sewage treatment had reduced Al-Ahli effluent TDS from 1885 mg/L to 880 mg/L, BOD from 943 mg/L to 440 mg/L, and total heavy-metal concentrations from 43 μg/L to 26 μg/L (Figure 4). EC had been reduced from 4.7 mmhos/cm to 3.9 mmhos/cm, dissolved CO_2_ from 888 mg/L to 599 mg/L, and COD from 4712 mg/L to 2200 mg/L (Table 2). The factory treatment had increased the pH from 5.9 to 6.4. The heavy-metal concentrations (μg/L) had been reduced as follows: Cr from 12 to 6, Pb from 8.2 to 5.9, Mn from 6 to 3.6, Cu from 8 to 5.9, and Cd from 9 to 5 (Table 2).

The treatments of the other three factories varied in their efficiency and depended on the parameter. Modern Leather Factory TDS was the lowest originally (672 mg/L) but it was not reduced by the sewage treatment. However, its BOD and COD were reduced to approximately half of the original. The lowest TDS (400 mg/L) and BOD (200 mg/L) values were reached in the treatment of Al Jabreen Leather Factory (Figure 4).

### 3.3. Tannery Wastewater Treatment Experiments

**Figure 5:**
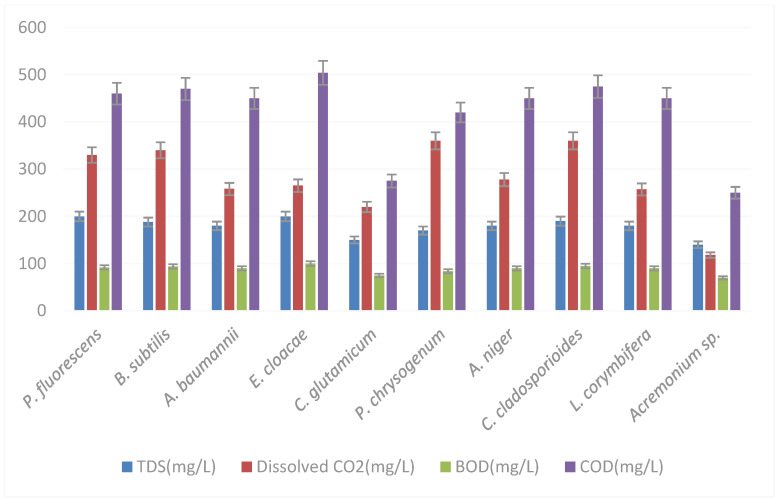
Pollution parameters (colors) (error bars for SD, *n* = 3) after the treatment of the factory-treated wastewater with single different bacteria and fungi. See the factory-treated concentrations (Al-Ahli in bold) in Table 2.

Five single bacterial and five single fungal species reduced the pollution parameters of the factory-treated Al-Ahli tannery wastewater, whose values are presented in Figure 4 and Table 2. The highest reduction was observed for *C. glutamicum* and *Acremonium* sp. from the factory-treated 880 mg/L TDS; *C. glutamicum* reduced TDS to 150 mg/L and *Acremonium* sp. to 140 mg/L (Figure 5).

**Figure 6:**
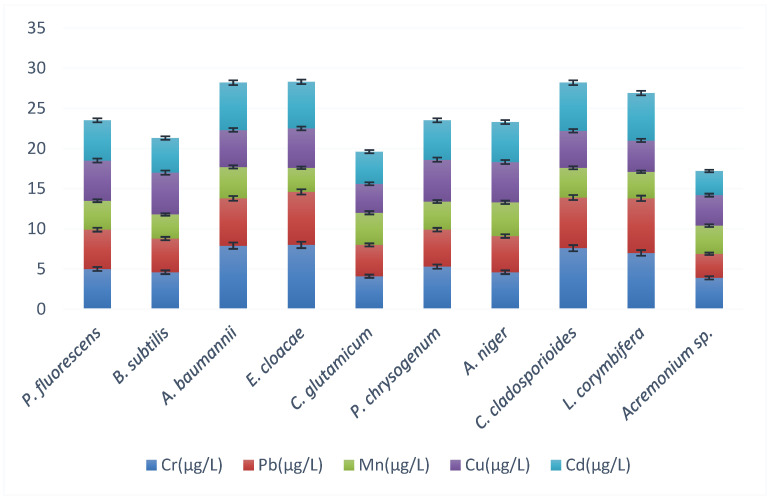
Heavy-metal concentrations (colors) (error bars for SD, *n* = 3) after the treatment of the factory-treated wastewater with single different bacteria and fungi. See the factory-treated concentrations (Al-Ahli in bold) in Table 2.

The respective values for dissolved CO_2_ were from 599 mg/L to 220 mg/L and 118 mg/L, BOD from 440 mg/L to 75 mg/L and 70 mg/L, and COD from 2200 mg/L to 275 mg/L and 250 mg/L (Figure 5). The total heavy-metal concentrations were reduced by *C. glutamicum* from the factory-treated 26 μg/L to 19.6 μg/L and by *Acremonium* sp. to 17 μg/L (Figure 6).

**Figure 7:**
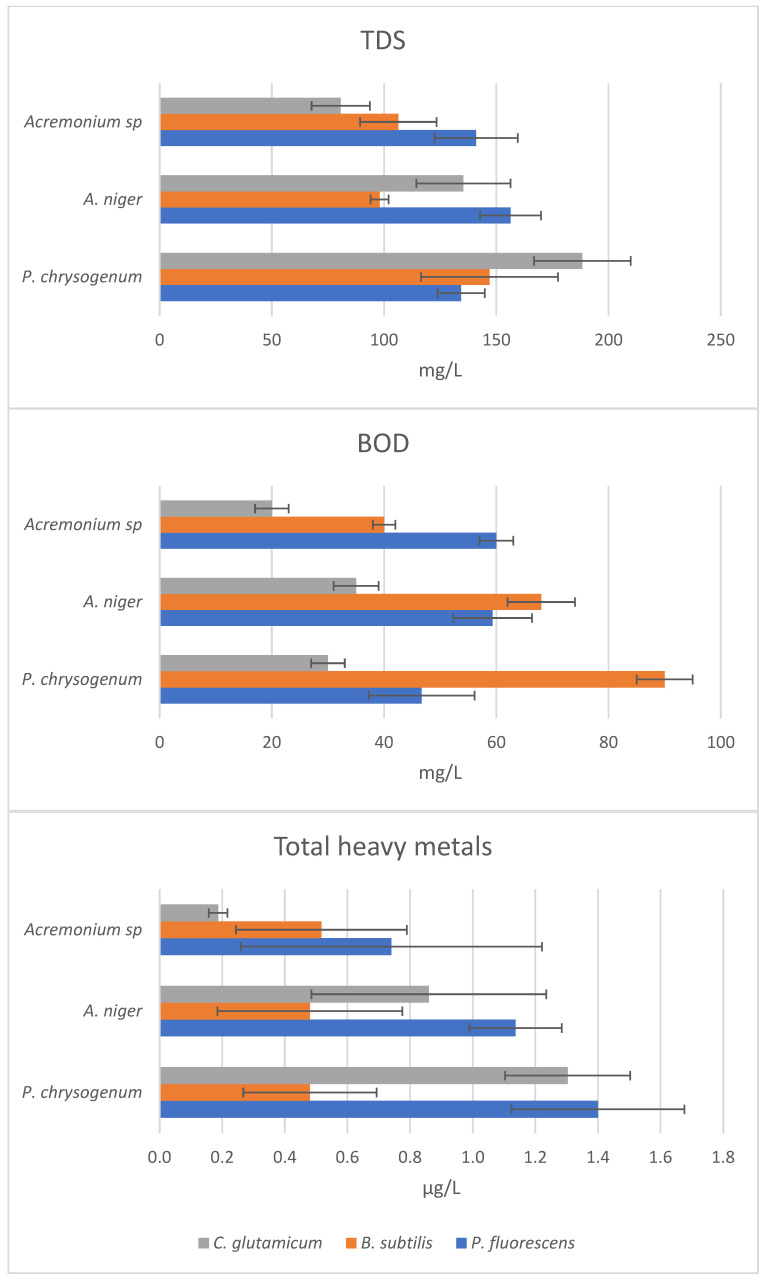
Pollution parameters (TDS, BOD, total heavy metals) after the treatment of the factory-treated wastewater with nine different consortia of bacteria (colors) and fungi (y-axis) (error bars for SD, *n* = 3). See the factory-treated concentrations in Figure 4.

The consortium of *C. glutamicum* and *Acremonium* sp. reduced the pollution parameters most. TDS was reduced from 880 to 80 mg/L and BOD from 440 to 20 mg/L (Figure 7).

COD was reduced from 2200 to 98 mg/L and dissolved CO_2_ from 599 mg/L to 270 mg/L (Table 3). Each single heavy-metal concentration was reduced remarkably by the consortium (Table 3).

The total heavy-metal concentration (the sum of metals) was reduced from the factory-treated value 26 μg/L to 0.2 μg/L (Figure 7). Each consortium reduced the collagen concentration from the factory-treated value of 120 mg/L. The highest reduction was observed for the consortium of *C. glutamicum* and *Acremonium* sp., which reduced collagen to about one-third (39 mg/L) (Table 3).

## 4. Discussion

Leather is produced in almost all countries in the world and the wastewater treatments in the tanneries vary considerably. While lacking in many developing countries, in industrial countries, the treatments can be efficient [35]. However, tanneries belong to the highly polluting industries throughout the world and pose a threat to many aquatic environments. Therefore, both cleaner processing practices and better wastewater treatments are needed [36,37]. Our sampling of tannery wastewaters revealed that all four tanneries in Saudi Arabia had failed to remove pollutants efficiently. Al-Ahli’s treatment removed one-third to half of the original pollutants. The total heavy-metal concentration was reduced by one-third and TDS, BOD, and COD by about half. Elsewhere, similar reductions (45–60%) of tannery effluents have been reported [38,39]. These reductions are not enough to satisfy the environmental regulations.

Regarding the environmental regulations, our biological treatment using the consortium of *C. glutamicum* and *Acremonium* sp. reduced almost all parameters below the limit for wastewater discharge to the environment in Saudi Arabia (environmental protection regulations, Saudi Arabia). It is notable that this consortium was the only treatment that succeeded in reducing most of the pollution parameters below their limits. The factory treatment of Al-Ahli reduced TDS to 880 mg/L, while the limit is 150 mg/L. The limit is almost double compared to the result of our best consortium, 80 mg/L. In comparison, the efficiency of the single species remained relatively poor; the single species *C. glutamicum* reduced TDS to 150 mg/L and *Acremonium* sp. to 140 mg/L. Thus, the consortium was far more efficient than the single species. This was the case for BOD as well. The limit for BOD is 25 mg/L. The factory treatment was far from acceptable, 440 mg/L. The single species treatments also remained at non-acceptable levels, 75 mg/L and 70 mg/L. The consortium treatment fell slightly below the limit, being 20 mg/L BOD. Moreover, the consortium reduced COD to 98 mg/L, while the limit is 100 mg/L. The only organic parameter that exceeded the limit was dissolved CO_2_. While the limit is 10 mg/L, our result was 270 mg/L. The heavy-metal concentrations were reduced below the limits, except in the case of Cd. The Cd concentration limit of 0.02 μg/L was slightly exceeded, being 0.03 µg/L. The limit for Cr is 0.1 μg/L and our consortium treatment reduced it to half of the limit, to 0.05 µg/L. The respective values for Pb are 0.1 μg/L vs. 0.02 µg/L, and for Cu, they are 0.2 µg/L vs. 0.02 µg/L. Comparing the consortium and single species, the total heavy-metal concentration was reduced to 19.6 μg/L by *C. glutamicum*, to 17 μg/L by *Acremonium* sp., and to 0.2 μg/L by their consortium. This shows the high efficiency of the consortium. As mentioned above, TDS and BOD were also more efficiently removed by the consortium than by the single species. These results verify the hypothesis: the consortium is more efficient in removing pollutants than the single species in the consortium. This is also true in the case of both the organic and inorganic pollutants studied. While each of the four factory treatments that take place in Saudi Arabia exceeded the limits for the wastewater pollutants greatly, our consortium treatment managed to reduce the pollutants below the limits in general.

Collagen and gelatin are present in tannery effluents [40]. Therefore, species that degrade these components are needed. Each of our consortia reduced the collagen concentration remarkably, and the most efficient consortium of *C. glutamicum* and *Acremonium* sp. reduced it to one-third of the original. For instance, *Pseudomonas aeruginosa* [41], *Bacillus* sp. [42,43], and *Actinobacteria* sp. [44] have been previously reported to produce gelatinase for the degradation of gelatin. Collagenase enzyme was shown to be produced also by *Bacillus cereus* [45], *Klebsiella pneumoniae* [46], *E. coli* [47], and the species of *Aspergillus*, *Cladosporium*, *Penicillium,* and *Alternaria* [48]. We can add all our species to the list and, as particularly efficient examples, *C. glutamicum* and *Acremonium* sp.

Tannery wastewaters need a combination of techniques to be treated adequately. The conventional techniques need a secondary biological treatment before the wastewater can be released into the environment [5]. The treatments used in the studied Saudi Arabian tanneries (DAF, activated sludge, and filtrations) were not efficient enough. More efficient biological treatment using, for instance, the consortium of microorganisms is needed and we propose here the consortium of the bacterium *C. glutamicum* and the fungus *Acremonium* sp. Previously, the consortia of several microbes (not identified) was shown to be efficient in cleaning tannery wastewater [28]. The consortium of the fungi *Cladosporium perangustum*, *Penicillium commune*, *Paecilomyces lilacinus*, and *Fusarium equiseti* was also used efficiently [49]. The fungi *Aspergillus niger* and *Penicillium* sp. were shown to reduce heavy metals from tannery wastewaters [50]. Heavy metals are known to be adsorbed also by bacteria, for instance, *Bacillus subtilis* and *B. megaterium* [50]. The bacterium we used, *C. glutamicum*, is a species that is studied actively for its bioactive compounds and tolerance against many different toxic compounds, such as azo dyes and heavy metals [51,52,53,54]. *C. glutamicum* biomass has been reported to adsorb, for instance, As and Pb [55,56]. In fact, *C. glutamicum* can be utilized in the bioremediation of many kinds of pollutants [57]. Our fungal species *Acremonium* sp. has also been studied for its bioremediation potential. *A. strictum* and *A. persicinum* have been shown to adsorb Cr and other heavy metals from water and mining soil [58,59]. High tolerance against heavy metals has been shown for *Acremonium* sp. when it is in a consortium with *B. subtilis* [25].

## 5. Conclusions

The consortium of *Corynebacterium glutamicum* and *Acremonium* sp. had the highest efficiency in reducing pollutants from tannery wastewater already treated by the factory. The consortium was more efficient than either of the single species, confirming the hypothesis presented here. This study provides a potential consortium for the biological treatment of tannery effluents to be studied further. We used real wastewater from the factory that certainly contained some microorganisms that we did not analyze. These microbes can vary from time to time and among tanneries, and can disturb the microbial cleaning process in some circumstances. Thus, more samples need to be studied from different tanneries to observe the variation. More studies are also needed regarding the final separation and treatment of the produced microbial biomass that contains heavy metals. The native microbes offer a cost-effective and eco-friendly treatment to clean tannery effluents and reduce the cytotoxic, genotoxic, and phytotoxic effects of tannery effluent discharged to the environment. The tanneries studied in Saudi Arabia need secondary treatment, which requires further study. In the arid climate of Saudi Arabia, it is also extremely valuable if the wastewater can be used in irrigation, which will be possible after the biological treatment proposed here.

## Figures and Tables

**Table 1:** Microorganisms with their NCBI accession numbers isolated from different sampling sites.

Isolate	Organism	Name	Accession Number
TWB1	Bacterium	*Pseudomonas fluorescens*	OR272078
TWB2	Bacterium	*Bacillus subtilis*	OR272079
TWB3	Bacterium	*Acinetobacter baumannii*	OR272080
TWB4	Bacterium	*Enterobacter cloacae*	OR272081
TWB5	Bacterium	*Corynebacterium glutamicum*	OR272082
TWF1	Fungus	*Penicillium chrysogenum*	OR294167
TWF2	Fungus	*Aspergillus niger*	OR290136
TWF3	Fungus	*Cladosporium cladosporioides*	OR294168
TWF4	Fungus	*Lichtheimia corymbifera*	OR295632
TWF5	Fungus	*Acremonium* sp.	OR294169

**Table 2:** Pollution parameters of different tannery effluents (mean ± SD, *n* = 3) before and after the factory sewage treatment. Bold values refer to the effluent used in the microbial treatment experiments.

Parameters	Sample Sites							
	Modern Leather Factory		Al Jabreen Leather Factory		Al-Ahli Leather Factory		Safi Universal for Leather Tanning	
	Before	After	Before	After	Before	After	Before	After
EC (mmhos/cm)	4.5 ± 2	3.6 ± 3	3.5 ± 1	2.8 ± 3	4.7 ± 1	**3.9 ± 2**	3.9 ± 2	2.9 ± 1
pH	6.2 ± 2	7.0 ± 3	6.5 ± 1	6.7 ± 2	5.9 ± 2	**6.4 ± 3**	6.5 ± 3	6.9 ± 2
CO_2_ (mg/L)	745 ± 88	465 ± 49	812 ± 68	486 ± 46	888 ± 98	**599 ± 47**	672 ± 25	478 ± 3
COD (mg/L)	3430 ± 99	1630 ± 98	2050 ± 78	1000 ± 90	4712 ± 130	**2200 ± 122**	2362 ± 123	1750 ± 78
Cr (μg/L)	10 ± 3	7.4 ± 2	9.4 ± 3	5 ± 2	12 ± 3	**6 ± 2**	10 ± 3	9 ± 2
Pb (μg/L)	8 ± 2	6.9 ± 3	5.2 ± 3	3 ± 3	8.2 ± 2	**5.9 ± 3**	8 ± 2	4 ± 3
Mn (μg/L)	7.8 ± 2	7.4 ± 2	11.2 ± 2	9.7 ± 3	6 ± 1	**3.6 ± 1**	7.2 ± 3	3.9 ± 4
Cu (μg/L)	8.2 ± 3	6 ± 3	8 ± 2	4.8 ± 1	8 ± 2	**5.9 ± 2**	7.7 ± 1	4 ± 1
Cd (μg/L)	6.8 ± 2	4.8 ± 4	6 ± 2	3.9 ± 1	9 ± 2	**5 ± 3**	9.2 ± 2	6.6 ± 2

**Table 3:** Properties (mean ± SD, *n* = 3) of the original tannery wastewater, factory-treated wastewater, and the factory-treated wastewater after it was treated with different consortia of one bacterium and one fungus. The most efficient appear in bold.

Bacterium	Fungus		
	*P. chrysogenum*	*A. niger*	*Acremonium* sp.
	Parameter	Original	Factory-treated
	EC mmhos/cm	4.7 ± 1	3.9 ± 2
*P. fluorescens*	1.6 ± 0.2	1.9 ± 0.1	1.6 ± 0.3
*B. subtilis*	1.2 ± 0.2	1.5 ± 0.3	1.3 ± 0.2
*C. glutamicum*	1.6 ± 0.1	1.2 ± 0.2	**1.0 ± 0.1**
	pH	5.9 ± 2	6.4 ± 3
*P. fluorescens*	7.2 ± 0.2	7.2 ± 0.1	7.1 ± 0.3
*B. subtilis*	7.2 ± 0.1	7.2 ± 0.1	7.3 ± 0.1
*C. glutamicum*	7.2 ± 0.1	7.1 ± 0.2	7.2 ± 0.2
	Dissolved CO_2_ (mg/L)	888 ± 98	599 ± 47
*P. fluorescens*	548 ± 32	520 ± 24	440 ± 13
*B. subtilis*	654 ± 56	580 ± 34	340 ± 25
*C. glutamicum*	488 ± 76	600 ± 35	**270 ± 32**
	COD (mg/L)	4712 ± 130	2200 ± 122
*P. fluorescens*	200 ± 12	220 ± 14	250 ± 8
*B. subtilis*	270 ± 19	290 ± 13	170 ± 17
*C. glutamicum*	199 ± 12	240 ± 78	**98 ± 15**
	Cr (μg/L)	12 ± 3	6 ± 2
*P. fluorescens*	0.9 ± 0.4	0.6 ± 0.4	0.9 ± 0.3
*B. subtilis*	0.3 ± 0.2	0.4 ± 0.1	0.2 ± 0.4
*C. glutamicum*	0.6 ± 0.2	0.8 ± 0.1	**0.05 ± 0.1**
	Pb (μg/L)	8.2 ± 2	5.9 ± 3
*P. fluorescens*	0.09 ± 0.01	0.07 ± 0.02	0.09 ± 0.01
*B. subtilis*	0.06 ± 0.04	0.03 ± 0.02	0.06 ± 0.02
*C. glutamicum*	0.05 ± 0. 1	0.03 ± 0. 2	**0.02 ± 0.2**
	Mn (μg/L)	6 ± 1	3.6 ± 1
*P. fluorescens*	0.6 ± 0.4	0.4 ± 0.04	0.09 ± 0.02
*B. subtilis*	**0.04 ± 0.03**	0.05 ± 0.02	**0.04 ± 0.02**
*C. glutamicum*	0.09 ± 0.03	0.08 ± 0.02	**0.04 ± 0.01**
	Cu (μg/L)	8 ± 2	5.9 ± 2
*P. fluorescens*	0.07 ± 0.02	0.07 ± 0.02	0.06 ± 0.02
*B. subtilis*	0.05 ± 0.04	0.06 ± 0.04	0.09 ± 0.03
*C. glutamicum*	0.5 ± 0.2	0.03 ± 0.01	**0.02 ± 0.02**
	Cd (μg/L)	9 ± 2	5 ± 3
*P. fluorescens*	**0.03 ± 0.02**	0.05 ± 0.03	**0.03 ± 0.01**
*B. subtilis*	0.07 ± 0.02	**0.03 ± 0.03**	0.08 ± 0.04
*C. glutamicum*	0.07 ± 0.02	0.09 ± 0.02	**0.03 ± 0.01**

## Data Availability

All data related to this manuscript are incorporated only into the manuscript.
